# Intravesical injection of peripheral blood mononuclear cell for the treatment of interstitial cystitis: A preliminary report

**DOI:** 10.1371/journal.pone.0324535

**Published:** 2025-05-21

**Authors:** Ching-Pei Tsai, Kai-Lun Cheng, Evelyn Yang, Lung-Yung Huang, Fu-Hui Wang, Man-Jung Hung, Hong-Lin Su

**Affiliations:** 1 Department of Life Sciences, National Chung Hsing University, Taichung, Taiwan; 2 Doctoral Program in Translational Medicine, National Chung Hsing University, Taichung, Taiwan; 3 Rong Hsing Translational Medicine Research Center, National Chung Hsing University, Taichung, Taiwan; 4 Department of Obstetrics and Gynecology, Taichung Veterans General Hospital, Taichung, Taiwan; 5 Department of Medical Imaging, Chung Shan Medical University Hospital, Taichung, Taiwan; 6 School of Medicine, Chung Shan Medical University, Taichung, Taiwan; 7 Department of Obstetrics and Gynecology, Chung Shan Medical University Hospital, Taichung, Taiwan; 8 Department of Obstetrics and Gynecology, School of Medicine, Colleague of Medicine, Chung Shan Medical University, Taichung, Taiwan; 9 Duogenic StemCells Corporation, Taichung, Taiwan; 10 The iEGG and Animal Biotechnology Research Center, National Chung Hsing University, Taichung, Taiwan; China Medical University, TAIWAN

## Abstract

Interstitial cystitis (IC) is a complex syndrome characterized by symptoms such as bladder pain, urgency, frequency, and nocturia, without the presence of urinary tract infection or any other identifiable pathology. Traditional treatments, including medication and bladder instillation, are often ineffective in about 30% of patients. Currently, efforts are focused on developing therapies based on the possible pathogenesis of IC. This study is a phase one clinical trial which aimed to investigate the safety and efficacy of autologous peripheral blood mononuclear cell (PBMC) intravesical injections, which have the potential to promote tissue regeneration, as a novel treatment for IC. The study involved isolating PBMCs using the Sepax Cell Separation System and injecting these cells beneath the bladder mucosa layer of patients with IC. Clinical efficacy was evaluated using voiding diaries, questionnaires, and cystoscopic examinations before and 3 months after treatment. Twelve patients with refractory IC participated in this study. Observed side effects, such as pain or urinary tract infection, were mild and transient which demonstrated the safety of this treatment modality. Although the treatment response varied among patients, a third of the patients experienced moderate to significant progress according to the GRA score. Four patients exhibited improvement in bladder glomerulations during postoperative follow-up cystoscopy. In conclusion, the overall safety profile of PBMCs injections appears to be favorable. Further research is needed to optimize treatment protocols and understand the factors influencing individual responses to this therapy.

## Introduction

Interstitial cystitis/bladder pain syndrome (IC/BPS) is a chronic (>6 weeks duration) pelvic condition that affects the urinary bladder with symptoms of discomfort, pressure, or pain. The condition is characterized by chronic inflammation and lower urinary tract symptoms, in the absence of urinary infections or any other identifiable pathologies [1–4]. Recent East Asian guidelines define IC as a bladder disease with Hunner lesions, usually associated with hypersensitive bladder (HSB) symptoms and bladder inflammation, and BPS as a condition with HSB symptoms in the absence of Hunner lesions and other possible etiologies [5].

Currently, the exact etiology of IC/BPS remains unclear and is often diagnosed after excluding other potential causes of these symptoms [2,4]. The probable cause is believed to be chronic bladder damage, with defects in the bladder mucosa being a common finding under cystoscopy. Treatment methods often employed include oral medication to alleviate symptoms and bladder instillation of drugs such as heparin or hyaluronic acid to repair the bladder mucosa. However, about 30% of patients feel that bladder instillation treatments are ineffective, and such treatments primarily offer symptomatic relief [6–8]. The long-term effectiveness and potential for cure remain unresolved [3,8]. This has led medical researchers to explore new treatment modalities of biologics, such as local injection of Botulinum ToxinA(BoNT-A), platelet-rich plasma (PRP), stromal vascular fraction, and mesenchymal stem cells [5,9–13]. Low energy shock wave therapy, which has anti-inflammatory and anti-apoptotic effects, has been reported with promising results for the treatment of IC [14]. BoNT-A is a safe treatment for IC/BPS patients, with a 41–52% rate of high to intermediate satisfaction [15]. However, a consistent conclusion of a positive effect cannot be drawn at the moment, as the published studies are small and heterogeneous in design [12]. Furthermore, postoperative urinary tract infections and voiding difficulty raise concerns regarding this treatment modality [16]. PRP, a regenerative medicine treatment, can repair the bladder epithelium and surrounding tissues through platelets and numerous regenerative factors [17–19]. However, current medical practice requires intensive treatment (monthly injections for four consecutive months), and its long-term effectiveness is yet to be ascertained [20,21].

Recent research has emphasized the role of M2 macrophages in down-regulating inflammatory responses, promoting angiogenesis, and aiding tissue repair [22–25]. CD14 + monocytes, which have the capability to differentiate into M2 macrophages, can be sourced from human umbilical cord blood [23] or peripheral blood mononuclear cells (PBMCs) [22,24]. In some clinical trials, PBMCs have shown similar clinical promise as mesenchymal stem cells in the disease treatment and tissue regeneration [26–30], including repeated implantation failure, limb ischemia, and wound repair in diabetic patients. Since PBMCs help reduce inflammation and contain precursors that stimulate and support the repair of tissue, they may have the potential to promote bladder tissue regeneration.

This study aims to explore the safety of intravesical PBMCs injections for IC/BPS. Despite the demonstrated potential efficacy of PBMCs in other conditions, the PBMCs application in IC/BPS, particularly through intravesical injection, has not yet been investigated. We hypothesized that bladder injection therapy using PBMCs might contribute to bladder tissue repair process and result in symptomatic and morphological improvement in patients with IC/BPS. In this study, the primary endpoint of this research is to assess the safety of intravesical PBMC injections in IC/BPS patients. In addition to safety, we aim to determine whether PBMCs provide therapeutic potential to address the unmet need in managing this chronic and debilitating disease.

## Materials and methods

### Participants

This study was approved by the Institutional Review Board and ethics committee of the Chung Shan Medical University Hospital (CS1–21167). Each patient was informed about the study rationale and procedures, and written, informed consent was obtained before treatment.

A total of 12 patients with IC/BPS, aged 20 and above, who had failed conventional treatments were enrolled in this study from May 2022 to March 2023 at the Department of Obstetrics and Gynecology, Chung Shan Medical University Hospital. The diagnosis of IC/BPS was based on characteristic symptoms, cystoscopic findings, and the ESSIC criteria [2]. All patients had previously undergone at least three types of treatment modalities, including lifestyle modifications, cystoscopic hydrodistention, non-steroidal anti-inflammatory drugs, oral pentosan polysulfate sodium or tricyclic antidepressants, intravesical instillation of hyaluronic acid, or intravesical botulinum toxin A injection, for a minimum of one year, but their symptoms either remained unchanged or relapsed [31]. The minimum time elapsed since the most recent botulinum toxin A injection was at least one year before this treatment. Patients who had previously received autologous blood mononuclear cell injection treatment, autologous platelet plasma bladder injection treatment, or those unable to cooperate with follow-ups were excluded from the study.

Each patient in the study underwent subjective and objective assessments of symptom severity both before treatment and three months after treatment. These assessments included bladder diary, the Interstitial Cystitis Symptom Index and Problem Index (ICSI and ICPI) and the Visual Analog Scale (VAS) for pain. Three months after the start of treatment, patients self-assessed their overall response using the Global Response Assessment (GRA). Cystoscopy was performed before treatment and three months after treatment, with findings recorded accordingly.

### Purification of peripheral blood mononuclear cells

For eligible participants, 100 mL of peripheral blood was drawn using a qualified blood bag containing an anticoagulant. The collected blood was then thoroughly mixed with 20 mL of MoFi cell culture medium with class II medical device certificate in Taiwan and USA. The mixture was left to stand for 30 minutes to accelerate the blood cell precipitation and PBMC enrichment. The isolated buffy coat of the blood was loaded on 100 mL of Ficoll-Paque Premium lymphocyte separation solution (Cytiva) to automatically separate the PBMCs population in Sepax II machine (Cytiva). The isolated PBMCs were then washed twice with 450 mL of saline to remove any residual medium and Ficoll-Paque before cell transplantation.

The final concentration of PBMCs was adjusted to 7–8 mL, containing approximately 5–15 × 10^7^ cells. A 1 mL sample was taken for identification and analysis at the Biomedical Industry R&D Center, Department of Life Sciences, Chung Hsing University, using a flow cytometer to identify the proportion of hematopoietic stem cells and monocytes. The remaining cells (total 6 ~ 7mL) were injected into the affected area.

The entire process from blood draw to automated mononuclear cell separation took approximately 2.5 hours. The separated blood sample was not stored for more than 2 hours to maintain cell viability. The cell purification process was conducted within a closed system to minimize the risk of contamination. The separation equipment was disposable and single-use, preventing cross-contamination of specimens. Before injection, a Luria Broth agar plate test was performed to count live bacteria and ensure no bacterial contamination in the cell sample. All twelve patients showed no bacterial contamination of their cell samples.

### Intravesical injection procedures

Autologous PBMCs are injected beneath the bladder mucosa layer of patients after they undergo bladder hydrodistension surgery. Under general anesthesia, an endoscopic injection needle (Cook® Williams Cystoscopic Injection Needle G14220 5Fr) was carefully inserted into the bladder submucosal space, targeting the posterior and lateral walls while avoiding the trigone area. For each injection site, 0.5 mL of PBMCs was administered, causing the bladder mucosa to bulge slightly. In each treatment session, the patients received a total of 6 ~ 7mL (12 ~ 14 shots) suburothelial injections. In most patients, injections of 0.5 mL PMBCs were administered equally throughout the bladder in four quadrants divided by the ureteral orifices (left lateral, left medial, right medial and right lateral quadrants). However, in patients with obvious bladder lesions, such as Hunner’s ulcers, the injections were targeted at the lesion sites. To prevent bacterial infection, patients receiving this cell therapy were prescribed a three-day course of antibiotics.

### Statistical analysis methods

Patient demographics, specifically age, body mass index (BMI), ESSIC type, glomerulation, were recorded. Continuous variables were expressed as medians and interquartile ranges (IQR), and categorical variables were presented as frequencies. The Wilcoxon’s matched pair signed rank test was used to compare changes in scores of GRA, ICSI, ICPI and VAS and cystoscopic capacity before and at the 3-month follow-up. Statistical analyses were performed using MedCalc Statistical Software version 20.008 (MedCalc Software, Ostend, Belgium; 2021). A *p* value of <0.05 was considered statistically significant.

## Results

All 12 patients with refractory interstitial cystitis completed one PBMCs injection. The median age of the patients was 52 years (IQR: 45.5-59.0 years), and the median BMI was 21.2 (IQR: 19.8-23.1). The duration of symptoms ranged from 2 to 10 years. All patients had previously received intravesical instillation of hyaluronic acid, and 4 patients had previously underwent intravesical botulinum toxin A injection. Blood draw reports are all within the normal range. The distribution of ESSIC types among the patients was as follows: ESSIC Type 2 in 9 patients and ESSIC Type 3 in 3 patients. Therefore, 3 patients with ulcerative IC were included in our study. Glomerulation scores varied, with 8 patients having a score of 1, 1 patients a score of 2, and 3 patients a score of 3 (Table 1).

**Table 1:** Patients’ characteristics.

Patient	Age	BMI	Duration (year)	Previous Therapy	Lab					ESSIC Typing	Glomerulation
					WBC (10^6^/L)	Hgb (g/dL)	PLT (10^9^/L)	CRE (mg/dL)	ALT (IU/L)		
1	38	22.4	4	HA, Botox	7050	12.7	295	0.60	13	2	3
2	61	20.3	5	HA, Botox	6390	13.0	392	0.92	20	2	1
3	50	21.6	4	HA	4580	12.4	188	0.54	21	3	1
4	55	19.7	4	HA, Botox	4090	12.4	222	0.80	18	2	3
5	57	26.3	3	HA	4980	12.7	173	0.59	18	3	1
6	67	19.5	10	HA	4740	12.6	189	0.95	38	3	1
7	50	19.7	7	HA, Botox	4680	13.3	253	0.79	15	2	1
8	54	20.8	10	HA	5010	12.6	196	0.62	14	2	3
9	27	19.8	2	HA	7580	12.2	318	0.60	8	2	1
10	45	25.8	2	HA	7090	12.7	351	0.66	27	2	1
11	46	21.6	4	HA	9040	13.2	260	0.53	12	2	1
12	62	23.9	4	HA	4960	14.7	192	0.64	14	2	2
Mean± SD	51.0 ± 11.1	21.8 ± 2.4	4.9 ± 2.7								

HA: Hyaluronic acid

**Fig 1:**
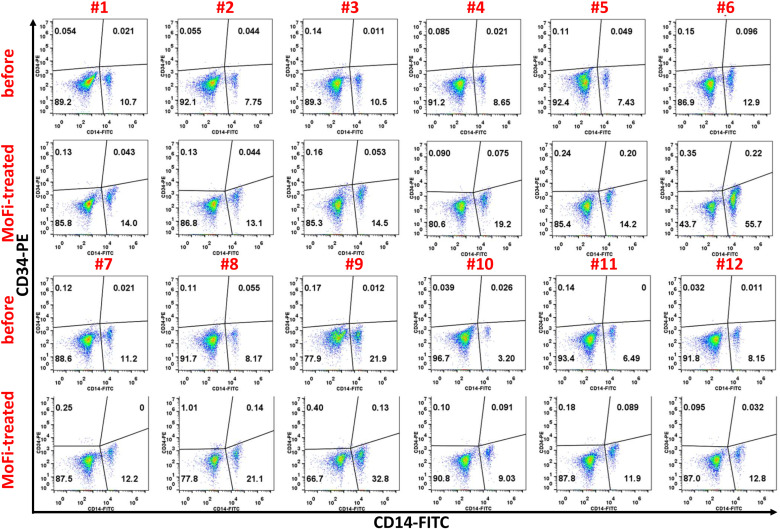
FACS Analysis of Monocytes Before and After Activation. The FACS (Fluorescence-Activated Cell Sorting) analysis of the monocytes (CD14: monocyte marker, CD206: M2 macrophage marker) before and after activation.

**Fig 2:**
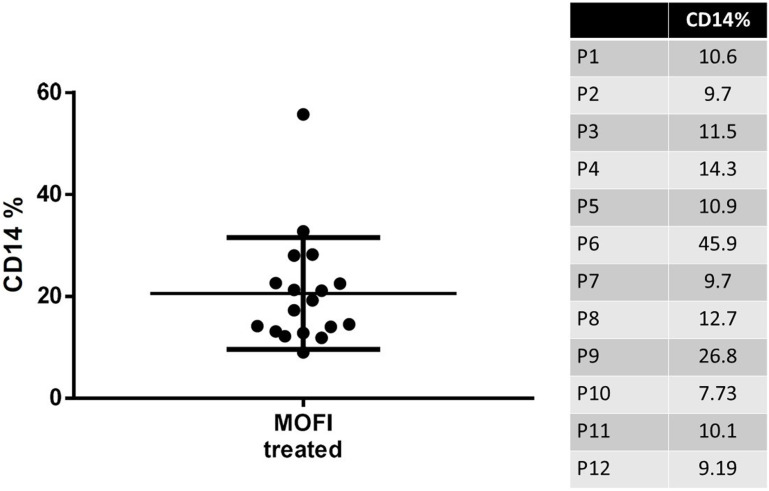
Proportion of CD14 ** + Monocytes Post-Purification.** The proportion of CD14 + monocytes after the separation and purification process.

On the day of the surgery, each patient underwent a blood draw of 100 ml, which was then processed using the Sepax II Cell Separation System to isolate PBMCs. The sterility of this peripheral blood cell purification (PCP) method was ensured by the closed tubing system, and no contamination of microbes during the cell processing was observed. The subpopulation ratios of the processed PBMCs were analyzed by flow cytometry. The FACS (Fluorescence-Activated Cell Sorting) analysis of the patients is shown in Fig 1. The proportion of CD14+ monocytes was approximately 10% or higher after the separation and purification process (Fig 2). The GMP-grade MoFi medium did not alter the characteristics of the isolated monocytes (CD14: monocyte marker), such as the differentiation potency of the cells into M2 macrophages.

Table 2 summarizes the adverse effects observed after the injection and during follow-up. Pain was reported by four patients (patients 1, 5, 10, and 11) within the first week post-operation. All patients’ pain improved after oral medication. Besides, one patient (patient 11) experienced urinary tract infections (UTIs), as indicated by a white blood cell count greater than 10 per high power field in urinalysis, in the first week post-operation. We prescribed this patient three days antibiotics and symptoms eased quickly. Final urine culture showed no growth of bacteria. Overall, no patients required additional hospitalization to manage complications related to this treatment, and no other discomfort was reported by any patients at the final follow-up.

**Table 2:** Post-injection Observations of Side Effects.

Patient No.	Pain	Hematuria	Fever	UTI	Voiding Difficulty	Others
1	Y	N	N	N	N	N
2	N	N	N	N	N	N
3	N	N	N	N	N	N
4	N	N	N	N	N	N
5	Y	N	N	N	N	N
6	N	N	N	N	N	N
7	N	N	N	N	N	N
8	N	N	N	N	N	N
9	N	N	N	N	N	N
10	Y	N	N	N	N	N
11	Y	N	N	Y	N	N
12	N	N	N	N	N	N

Table 3 presents the clinical data assessment results before and after surgery for these patients. The results indicated no significant difference in the severity of symptoms before and after treatment regarding ICSI, ICPI, VAS scores, and bladder dairy. The median Global Response Assessment (GRA) score post-treatment was 0.5 (IQR: -1.5 to 2.0). A third of the patients (4/12, 33%) reported GRA scores of +2 to +3 which indicated moderate-marked improvement in symptoms while 5 patients (5/12, 42%) reported GRA scores of 0 to +1 which indicated a stable disease. Three patients (3/12, 25%) reported GRA scores of -1 to -3. Increases in average voided volume (79.9 mL to 104 mL) and functional bladder capacity (145.8 mL to 184 mL) were found according to three-day voiding diary, but these changes did not reach statistical significance.

**Table 3:** Treatment responses with bladder injection therapies using PBMCs.

	GRA	ICSI		ICPI			VAS		VV		FBC		Glomerulation		Cystoscopic capacity	
Patient	Post-op	Pre-op	Post-op	Pre-op		Post-op	Pre-op	Post-op	Pre-op	Post-op	Pre-op	Post-op	Pre-op	Post-op	Pre-op	Post-op
1	-2	17	19	12		14	5	6	63.4	56.2	120	120	3	3	750	750
2	+2	3	2	2		2	0	0	72.1	161.1	110	350	1	1	1000	1000
3	+3	2	4	2		3	0	3	102.0	200.5	125	270	1	0	750	750
4	0	16	16	15		15	5	5	34.6	74.4	100	200	3	2	950	800
5	0	9	12	12		11	7	7	51.9	54.4	85	80	1	1	450	450
6	+1	15	10	12		8	6	3	75.8	97.3	160	180	1	1	500	500
7	+2	11	8	10		8	8	6	28.9	38.8	100	120	1	1*	950	950
8	+2	15	4	12		8	8	0	47.8	57.9	80	120	3	N.D	600	N.D
9	+1	14	12	13		12	5	5	135.3	200.0	240	250	1	1#	750	750
10	-1	3	6	3		7	0	4	98.0	81.0	190	120	1	N.D	500	N.D
11	-3	6	15	8		14	5	7	131.7	94.4	240	180	1	2	650	650
12	+1	11	6	7		3	3	1	116.8	131.7	200	220	2	2	650	650
Average	0.5	10.2 ± 5.5	9.5 ± 5.4	9.0 ± 4.6		8.8 ± 4.5	4.3 ± 3.0	3.9 ± 2.5	79.9 ± 36.6	104.0 ± 56.6	145.8 ± 58.3	184.2 ± 78.7			708.3 ± 185.7	725.0 ± 173.6
*p value*		*0.66*			*0.79*		*0.66*		*0.07*		*0.15*				*0.34 (n = 10)*	

Voided Volume (VV) is the average voided volume, and Functional Bladder Capacity (FBC) is the maximum urine volume, both recorded in the three-day voiding diary. N.D: no data available. *:less than 10% glomerulation area. #:less than 20% glomerulation area.

**Fig 3:**
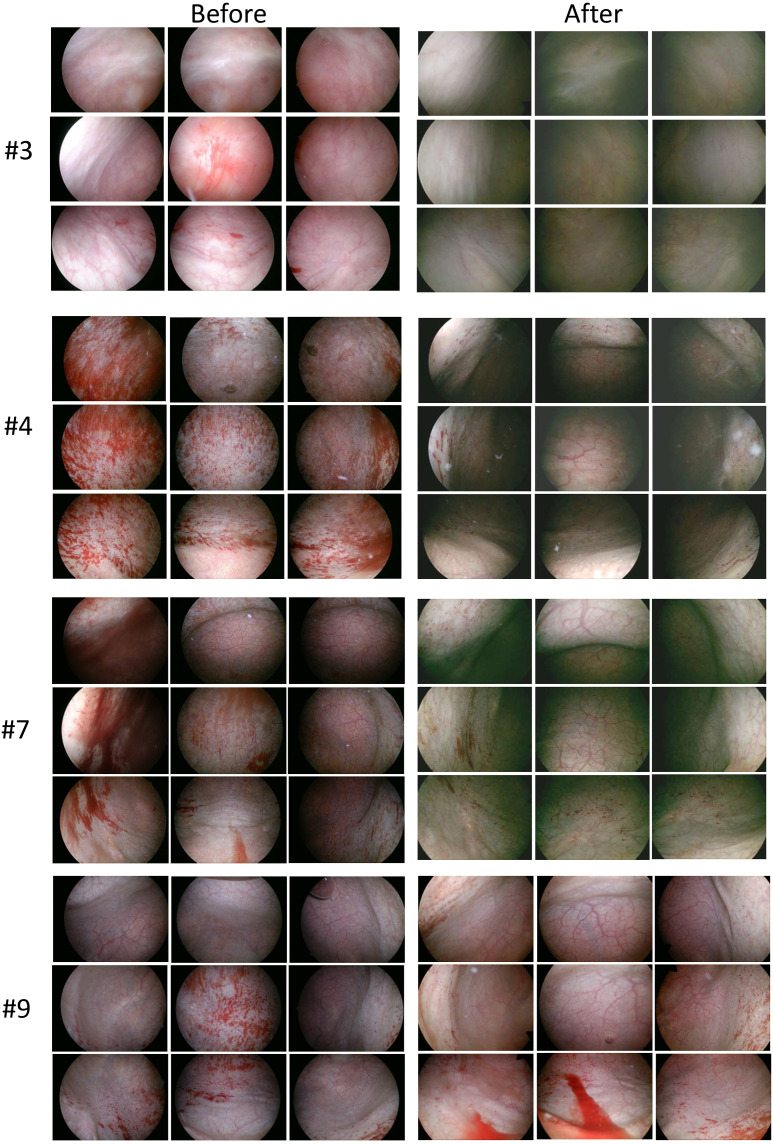
Cystoscopic Findings Pre- and Post-PBMC Injection. Cystoscopic findings before and 3 months after PBMCs intravesical injection in cases 3, 4, 7 and 9.

During the follow-up cystoscopy three months after surgery, no abnormal bladder lesions were detected. In terms of the change of glomerulations after hydrodistension, most patients remain unchanged (table 3), while some patients (4/12, 33%) demonstrated a notable improvement after the surgery (Fig 3). Among case 3,4,7,9, the surface area of glomerulation distribution is much smaller.

## Discussion

In this study, we evaluated the safety and efficacy of autologous PBMCs injections for treating patients with refractory interstitial cystitis (IC). The results indicate that while some patients experienced temporary side effects, the overall treatment was deemed safe. Importantly, no patients required additional hospitalization to manage complications related to the treatment, and no obvious discomfort was reported by any patients at the final follow-up. However, patient responses varied, potentially due to the complexity of their clinical conditions, highlighting the need for a more tailored approach in IC patients. We believe that this pilot, phase I clinical study showcases the safety of this intervention and further studies will be necessary to further elucidate the therapeutic effect.

PBMCs have been used to treat repeated implantation failure, intervertebral disc degeneration [26], knee osteoarthritis [27], diabetic foot [29,30], and amyotrophic lateral sclerosis [28] with initial results. In our previous report [27], we demonstrated that only the MoFi-treated PBMNCs, rather than the saline-treated PBMNCs, attenuated the joint inflammation and swelling in complete Freund’s adjuvant (CFA)-triggered inflammatory arthritis in rats. We further demonstrated that this anti-inflammatory potency was similar to that of bone marrow-derived mesenchymal stem cells. In an intervertebral disc degeneration (IVDD) rat model [26], we provided the in vitro and preclinical evidence in rats, showing that the MoFi-treated PBMCs exhibited anti-inflammatory and moderate tissue-repair effects on controlling IVDD progress in the rat model. The PBMCs significantly steered the aggrecan and type II collagen expressions and attenuated the pro-inflammatory cytokines in the affected discs. These results suggest that transient incubation with MoFi medium is necessary for the isolated PBMNCs to control the inflammation and may promote the monocytes toward the M2 macrophage differentiation in vivo. Here, we present the first case series to employ PBMCs for treating refractory interstitial cystitis. These findings suggest that PBMCs injection is a potentially safe therapeutic option for IC patients, showing no significant or lasting adverse effects.

The pain experienced by four patients within the first week post-operation could be due to a variety of factors. One potential explanation is the use of anticoagulants containing citrate during the preparation of mononuclear cells. When these PBMCs are injected into the bladder submucosal space, the citrate may intensify the pain. In patients with IC, inflammatory regions are accompanied by disrupted bladder barrier function, and increased urothelial permeability [32], which may sensitize the citrate-triggered pain sensations and intensify the sensory nerve fibers in the urothelium [33]. Furthermore, clinical evaluation scales showed that the scores for these four patients increased after treatment. The occurrence of pain within the first-week post-operation may serve as a clinical indicator of poor treatment efficacy in the future. Although some patients experienced pain, it was completely alleviated after analgesic medication. Therefore, it is likely that the flare-ups were due to the irritation of injection therapy rather than from the PBMCs themselves. These side effects are manageable and acceptable in the clinical context.

In addition to pain, one patient (No. 11) developed a urinary tract infection (UTI) within one-week post-operation. Previous studies have confirmed that UTIs are a potential risk associated with intravesical injections [9,34–40]. For instance, botulinum toxin A injections have been reported to result in UTI rates ranging from 6.7% to 15.4% [34,36], although other studies suggest that while the risk of UTI is present, it is relatively low [9,35,37,38,40]. Nonetheless, these data collectively highlight that UTI is indeed a potential complication. Despite administering a three-day course of antibiotics to all patient’s post-operation in the present study, the occurrence of UTIs remains a potential risk. These findings underscore the importance of monitoring for UTIs following PBMCs injections, as well as the need for careful consideration of antibiotic prophylaxis. While the overall safety profile of PBMCs injections appears favorable, the possibility of such complications highlights the need for continued vigilance in post-operative care and further improvement in optimizing treatment protocols to minimize adventitious infection.

Currently, no effective and long-lasting treatments for interstitial cystitis (IC) exist, and ongoing efforts are focused on developing and testing therapies based on the possible pathogenesis of IC. The application of adult and pluripotent stem cells in animal models of IC has been extensively evaluated [41], leading to biologics treatments such as intravesical injection with mesenchymal stem cells [10] or nanofat [11], which have shown preliminary positive results in IC patients. PBMCs are rich in CD14^+^ monocytes, which have potential therapeutic effects for IC. In our study, although the treatment effects did not reach statistical significance, some patients demonstrated improvements in GRA, average voided volume, functional bladder capacity, glomerulations and cystoscopic capacity after PBMCs injection. Overall average scores for ICSI, ICPI and pain-VAS were also improved. Four patients reported GRA scores of moderate-marked improvements in their symptoms while five patients exhibited stable disease status post-injection. The improvements suggest a tissue repair and regeneration therapeutic effect of the PBMCs. However, our patients received only a single treatment, which raises the question of whether multiple treatments might enhance the therapeutic outcome. In future studies, we plan to explore the potential benefits of repeated PBMCs injections, the selection of patients with milder IC symptoms, and the role of CD14^+^ cell proportion in strengthening the therapeutic effects.

This study has several limitations that should be acknowledged. First, the relatively small sample size of 12 patients may limit the generalizability of the findings for supporting significant differences in treatment outcomes. Additionally, the short follow-up duration of three months may also be insufficient to capture long-term effects or potential late-onset side effects of the treatment. Furthermore, the absence of a control group makes it difficult to ascertain the true efficacy of PBMCs injections compared to other treatments and the potential for placebo effects. The variability in patient responses observed in this study, potentially due to the complexity of their clinical conditions, complicates the interpretation of the overall results. Further research is necessary, including the need for larger sample sizes, longer follow-up periods, and the recruitment of control groups to better assess the efficacy and safety of PBMC injections on treating interstitial cystitis.

## Conclusions

This study provides preliminary evidence supporting the safety of autologous PBMCs injections in the treatment of refractory interstitial cystitis (IC). Although the treatment did not achieve statistically significant improvements in clinical outcomes, it was shown to be safe to use on IC patients. The observed side effects, including transient pain and urinary tract infections, were manageable with standard symptomatic treatment and did not necessitate additional hospitalization. These findings underscore the need for further research to explore the suitable regimen for PMBCs preparation, the efficacy of repeated PBMCs injections, the selection of appropriate patient populations, and the optimization of CD14^+^ cell numbers and proportions to enhance therapeutic outcomes. As one of the first case series to investigate PBMCs therapy for IC, this study lays the groundwork for future clinical trials that might consolidate PBMCs injections as a viable treatment option for this challenging condition.

## References

[1] Hanno PM, Landis JR, Matthews-Cook Y, Kusek J, Nyberg L Jr. The diagnosis of interstitial cystitis revisited: lessons learned from the National Institutes of Health Interstitial Cystitis Database study. J Urol. 1999;161(2):553–7. doi: 10.1016/s0022-5347(01)61948-7 9915447

[2] van de Merwe JP, Nordling J, Bouchelouche P, Bouchelouche K, Cervigni M, Daha LK, et al. Diagnostic criteria, classification, and nomenclature for painful bladder syndrome/interstitial cystitis: an ESSIC proposal. Eur Urol. 2008;53(1):60–7. doi: 10.1016/j.eururo.2007.09.019 17900797

[3] Moldwin RM, Sant GR. Interstitial cystitis: a pathophysiology and treatment update. Clin Obstet Gynecol. 2002;45(1):259–72. doi: 10.1097/00003081-200203000-00027 11862078

[4] Rahnama’i MS, Javan A, Vyas N, Lovasz S, Singh N, Cervigni M, et al. Bladder Pain Syndrome and Interstitial Cystitis Beyond Horizon: Reports from the Global Interstitial Cystitis/Bladder Pain Society (GIBS) Meeting 2019 Mumbai - India. Anesth Pain Med. 2020;10(3):e101848. doi: 10.5812/aapm.101848 32944561 PMC7472163

[5] Lin C-C, Huang Y-C, Lee W-C, Chuang Y-C. New Frontiers or the Treatment of Interstitial Cystitis/Bladder Pain Syndrome - Focused on Stem Cells, Platelet-Rich Plasma, and Low-Energy Shock Wave. Int Neurourol J. 2020;24(3):211–21. doi: 10.5213/inj.2040104.052 33017892 PMC7538293

[6] Hung M-J, Tsai C-P, Lin Y-H, Huang W-C, Chen G-D, Shen P-S. Hyaluronic acid improves pain symptoms more than bladder storage symptoms in women with interstitial cystitis. Taiwan J Obstet Gynecol. 2019;58(3):417–22. doi: 10.1016/j.tjog.2018.11.033 31122535

[7] Tsai C-P, Yang J-M, Liang S-J, Lin Y-H, Huang W-C, Lin T-Y, et al. Factors associated with treatment outcomes after intravesical hyaluronic acid therapy in women with refractory interstitial cystitis: A prospective, multicenter study. J Chin Med Assoc. 2021;84(4):418–22. doi: 10.1097/JCMA.0000000000000498 33784267 PMC12966032

[8] Fall M, Oberpenning F, Peeker R. Treatment of bladder pain syndrome/interstitial cystitis 2008: can we make evidence-based decisions?. Eur Urol. 2008;54(1):65–75. doi: 10.1016/j.eururo.2008.03.086 18403099

[9] Jiang Y-H, Jhang J-F, Kuo H-C. The clinical application of intravesical botulinum toxin A injection in patients with overactive bladder and interstitial cystitis. Tzu Chi Medical Journal. 2023;35(1). 10.4103/tcmj.tcmj_313_21 PMC9972932 36866354

[10] Shin JH, Ryu C-M, Yu HY, Park J, Kang AR, Shin JM, et al. Safety of Human Embryonic Stem Cell-derived Mesenchymal Stem Cells for Treating Interstitial Cystitis: A Phase I Study. Stem Cells Transl Med. 2022;11(10):1010–20. doi: 10.1093/stcltm/szac065 36069837 PMC9585946

[11] Hung M-J, Tsai C-P, Ying T-H, Chen G-D, Su H-L, Tseng C-J. Improved symptoms and signs of refractory interstitial cystitis in women after intravesical Nanofat plus platelet-rich plasma grafting: A pilot study. J Chin Med Assoc. 2022;85(6):730–5. doi: 10.1097/JCMA.0000000000000735 35507021 PMC12755650

[12] Rahnama’i MS, Marcelissen T, Apostolidis A, Veit-Rubin N, Schurch B, Cardozo L, et al. The efficacy of botulinum toxin A and sacral neuromodulation in the management of interstitial cystitis (IC)/bladder pain syndrome (BPS), what do we know? ICI-RS 2017 think thank, Bristol. Neurourol Urodyn. 2018;37(S4):S99–107. doi: 10.1002/nau.23493 29363792

[13] Rahnama’i MS, Salehi-Pourmehr H, Saeedi S, Tayebi S, Hajebrahimi S. Intravesical injection of abobotulinumtoxin-A in patients with bladder pain syndrome/interstitial cystitis. Urol Res Pract. 2023;49(3):205. 37877871 10.5152/tud.2023.22243 PMC10346116

[14] Shen Y-C, Tyagi P, Lee W-C, Chancellor M, Chuang Y-C. Improves symptoms and urinary biomarkers in refractory interstitial cystitis/bladder pain syndrome patients randomized to extracorporeal shock wave therapy versus placebo. Sci Rep. 2021;11(1):7558. doi: 10.1038/s41598-021-87040-1 33824389 PMC8024394

[15] Rahnama’i M-S, Salehi-Pourmehr H, Saeedi S, Tayebi S, Hajebrahimi S. Intravesical Injection of Abobotulinumtoxin-A in Patients with Bladder Pain Syndrome/Interstitial Cystitis. Urol Res Pract. 2023;49(3):205–10. doi: 10.5152/tud.2023.22243 37877871 PMC10346116

[16] Meng E, Hsu Y-C, Chuang Y-C. Advances in intravesical therapy for bladder pain syndrome (BPS)/interstitial cystitis (IC). Low Urin Tract Symptoms. 2018;10(1):3–11. doi: 10.1111/luts.12214 29341502

[17] Kwapisz A, Prabhakar S, Compagnoni R, Sibilska A, Randelli P. Platelet-Rich Plasma for Elbow Pathologies: a Descriptive Review of Current Literature. Curr Rev Musculoskelet Med. 2018;11(4):598–606. doi: 10.1007/s12178-018-9520-1 30255288 PMC6220004

[18] Ke Q-S, Jhang J-F, Lin T-Y, Ho H-C, Jiang Y-H, Hsu Y-H, et al. Therapeutic potential of intravesical injections of platelet-rich plasma in the treatment of lower urinary tract disorders due to regenerative deficiency. Tzu Chi Med J. 2019;31(3):135–43. doi: 10.4103/tcmj.tcmj_92_19 31258287 PMC6559029

[19] Dönmez Mİ, İnci K, Zeybek ND, Doğan HS, Ergen A. The Early Histological Effects of Intravesical Instillation of Platelet-Rich Plasma in Cystitis Models. Int Neurourol J. 2016;20(3):188–96. doi: 10.5213/inj.1632548.274 27706013 PMC5083831

[20] Jhang J-F, Wu S-Y, Lin T-Y, Kuo H-C. Repeated intravesical injections of platelet-rich plasma are effective in the treatment of interstitial cystitis: a case control pilot study. Low Urin Tract Symptoms. 2019;11(2):O42–7. doi: 10.1111/luts.12212 29265766

[21] Jiang Y-H, Jhang J-F, Lin T-Y, Ho H-C, Hsu Y-H, Kuo H-C. Therapeutic Efficacy of Intravesical Platelet-Rich Plasma Injections for Interstitial Cystitis/Bladder Pain Syndrome-A Comparative Study of Different Injection Number, Additives and Concentrations. Front Pharmacol. 2022;13:853776. doi: 10.3389/fphar.2022.853776 35392571 PMC8980355

[22] Chernykh ER, Shevela EY, Starostina NM, Morozov SA, Davydova MN, Menyaeva EV, et al. Safety and Therapeutic Potential of M2 Macrophages in Stroke Treatment. Cell Transplant. 2016;25(8):1461–71. doi: 10.3727/096368915X690279 26671426

[23] Marchetti V, Yanes O, Aguilar E, Wang M, Friedlander D, Moreno S, et al. Differential macrophage polarization promotes tissue remodeling and repair in a model of ischemic retinopathy. Sci Rep. 2011;1:76. doi: 10.1038/srep00076 22355595 PMC3216563

[24] Chernykh ER, Shevela EY, Sakhno LV, Tikhonova MA, Petrovsky YL, Ostanin AA. The generation and properties of human M2-like macrophages: potential candidates for CNS repair. Cell Ther Transplant. 2010;2(6):1–8.

[25] Gordon S, Taylor PR. Monocyte and macrophage heterogeneity. Nat Rev Immunol. 2005;5(12):953–64. doi: 10.1038/nri1733 16322748

[26] Chung Y-H, Hu M-H, Kao S-C, Kao Y-H, Wang F-H, Hsieh C-Y, et al. Preclinical Animal Study and Pilot Clinical Trial of Using Enriched Peripheral Blood-Derived Mononuclear Cells for Intervertebral Disc Degeneration. Cell Transplant. 2024;33:9636897231219733. doi: 10.1177/09636897231219733 38173231 PMC10768619

[27] Chuang C-H, Kuo C-C, Chiang Y-F, Lee P-Y, Wang F-H, Hsieh C-Y, et al. Enriched Peripheral Blood-Derived Mononuclear Cells for Treating Knee Osteoarthritis. Cell Transplant. 2023;32:9636897221149445. doi: 10.1177/09636897221149445 36661223 PMC9903009

[28] Li X-Y, Liang Z-H, Han C, Wei W-J, Song C-L, Zhou L-N, et al. Transplantation of autologous peripheral blood mononuclear cells in the subarachnoid space for amyotrophic lateral sclerosis: a safety analysis of 14 patients. Neural Regen Res. 2017;12(3):493–8. doi: 10.4103/1673-5374.202918 28469667 PMC5399730

[29] Ragghianti B, Berardi BM, Mannucci E, Monami M. Autologous Peripheral Blood Mononuclear Cells in Patients with Small Artery Disease and Diabetic Foot Ulcers: Efficacy, Safety, and Economic Evaluation. J Clin Med. 2023;12(12):4148. doi: 10.3390/jcm12124148 37373842 PMC10298945

[30] Meloni M, Giurato L, Andreadi A, Bellizzi E, Bellia A, Lauro D, et al. Peripheral Blood Mononuclear Cells: A New Frontier in the Management of Patients with Diabetes and No-Option Critical Limb Ischaemia. J Clin Med. 2023;12(19):6123. doi: 10.3390/jcm12196123 37834766 PMC10573900

[31] Jhang J-F, Lin T-Y, Kuo H-C. Intravesical injections of platelet-rich plasma is effective and safe in treatment of interstitial cystitis refractory to conventional treatment-A prospective clinical trial. Neurourol Urodyn. 2019;38(2):703–9. doi: 10.1002/nau.23898 30576011

[32] Graham E, Chai TC. Dysfunction of bladder urothelium and bladder urothelial cells in interstitial cystitis. Curr Urol Rep. 2006;7(6):440–6. doi: 10.1007/s11934-006-0051-8 17052438

[33] Parsons CL. The role of a leaky epithelium and potassium in the generation of bladder symptoms in interstitial cystitis/overactive bladder, urethral syndrome, prostatitis and gynaecological chronic pelvic pain. BJU Int. 2011;107(3):370–5. doi: 10.1111/j.1464-410X.2010.09843.x 21176078

[34] Jhang J-F, Yu W-R, Kuo H-C. Comparison of the Clinical Efficacy and Adverse Events between Intravesical Injections of Platelet-Rich Plasma and Botulinum Toxin A for the Treatment of Interstitial Cystitis Refractory to Conventional Treatment. Toxins (Basel). 2023;15(2):121. doi: 10.3390/toxins15020121 36828435 PMC9961286

[35] Giannantoni A, Gubbiotti M, Bini V. Botulinum Neurotoxin A Intravesical Injections in Interstitial Cystitis/Bladder Painful Syndrome: A Systematic Review with Meta-Analysis. Toxins (Basel). 2019;11(9):510. doi: 10.3390/toxins11090510 31480323 PMC6784147

[36] Kuo Y-C, Kuo H-C. Adverse Events of Intravesical OnabotulinumtoxinA Injection between Patients with Overactive Bladder and Interstitial Cystitis--Different Mechanisms of Action of Botox on Bladder Dysfunction?. Toxins (Basel). 2016;8(3):75. doi: 10.3390/toxins8030075 26999201 PMC4810220

[37] Kuo H-C, Jiang Y-H, Tsai Y-C, Kuo Y-C. Intravesical botulinum toxin-A injections reduce bladder pain of interstitial cystitis/bladder pain syndrome refractory to conventional treatment - A prospective, multicenter, randomized, double-blind, placebo-controlled clinical trial. Neurourol Urodyn. 2016;35(5):609–14. doi: 10.1002/nau.22760 25914337

[38] Akiyama Y, Nomiya A, Niimi A, Yamada Y, Fujimura T, Nakagawa T, et al. Botulinum toxin type A injection for refractory interstitial cystitis: A randomized comparative study and predictors of treatment response. Int J Urol. 2015;22(9):835–41. doi: 10.1111/iju.12833 26041274

[39] Ginsberg D, Gousse A, Keppenne V, Sievert K-D, Thompson C, Lam W, et al. Phase 3 efficacy and tolerability study of onabotulinumtoxinA for urinary incontinence from neurogenic detrusor overactivity. J Urol. 2012;187(6):2131–9. doi: 10.1016/j.juro.2012.01.125 22503020

[40] Tirumuru S, Al-Kurdi D, Latthe P. Intravesical botulinum toxin A injections in the treatment of painful bladder syndrome/interstitial cystitis: a systematic review. Int Urogynecol J. 2010;21(10):1285–300. doi: 10.1007/s00192-010-1162-9 20449567

[41] Dayem AA, Kim K, Lee SB, Kim A, Cho S-G. Application of Adult and Pluripotent Stem Cells in Interstitial Cystitis/Bladder Pain Syndrome Therapy: Methods and Perspectives. J Clin Med. 2020;9(3):766. doi: 10.3390/jcm9030766 32178321 PMC7141265

